# The Barley Chloroplast Mutator (*cpm*) Mutant: All Roads Lead to the *Msh*1 Gene

**DOI:** 10.3390/ijms23031814

**Published:** 2022-02-05

**Authors:** Franco Lencina, Alejandra Landau, Alberto R. Prina

**Affiliations:** Instituto de Genética “Ewald A. Favret”, CICVyA (Centro de Investigación en Ciencias Veterinarias y Agronómicas), INTA (Instituto Nacional de Tecnología Agropecuaria), Dr. Nicolás Repetto y de los Reseros s/n, Hurlingham 1686, Argentina; landau.alejandra@inta.gob.ar (A.L.); prina.albertoraul@inta.gob.ar (A.R.P.)

**Keywords:** mismatch repair, barley chloroplast mutator, plastome instability, *Msh*1 gene

## Abstract

The barley chloroplast mutator (*cpm*) is a nuclear gene mutant that induces a wide spectrum of cytoplasmically inherited chlorophyll deficiencies. Plastome instability of *cpm* seedlings was determined by identification of a particular landscape of polymorphisms that suggests failures in a plastome mismatch repair (MMR) protein. In Arabidopsis, *MSH* genes encode proteins that are in charge of mismatch repair and have anti-recombination activity. In this work, barley homologs of these genes were identified, and their sequences were analyzed in control and *cpm* mutant seedlings. A substitution, leading to a premature stop codon and a truncated MSH1 protein, was identified in the *Msh*1 gene of *cpm* plants. The relationship between this mutation and the presence of chlorophyll deficiencies was established in progenies from crosses and backcrosses. These results strongly suggest that the mutation identified in the *Msh*1 gene of the *cpm* mutant is responsible for the observed plastome instabilities. Interestingly, comparison of mutant phenotypes and molecular changes induced by the barley *cpm* mutant with those of Arabidopsis *MSH*1 mutants revealed marked differences.

## 1. Introduction

Plastids and mitochondria are plant cytoplasmic organelles that are considered semi-autonomous in the sense that they only encode part of the proteins they need. Most plastidial and mitochondrial proteins are nuclear-encoded, synthesized in the cytosol and imported with the help of dedicated translocation complexes. Organelle genomes differ from nuclear ones not only in their genetic behavior but also the mechanisms of DNA replication and maintenance [1,2,3,4].

With some exceptions, organellar genomes usually behave more conservatively than nuclear genomes in terms of mutation rates [5,6,7,8,9,10]. For this reason, experimental materials carrying mutations in nuclear genes controlling organelle genome stability are useful tools for expanding the scarce cytoplasmic variability that is available for research and/or plant breeding [11,12,13,14,15].

Several such cases have been detected based on the presence of clonally variegated plants and their breeding behavior in reciprocal crosses [11,16]. One of them is the barley chloroplast mutator (*cpm*) mutant, which was described as inducing a wide spectrum of cytoplasmically inherited chlorophyll mutants. This is in contrast with several examples in barley, maize and rice inducing a narrow spectrum of maternally inherited chlorophyll mutants, which also differed in showing a faster sorting out [17]. The first evidence of genetic instability in this experimental material was supplied as longitudinal stripes of diverse colors and sizes in plants of the fourth and fifth generation after a seed mutagenic treatment. Afterwards, cytoplasmic inheritance of the chlorophyll deficiencies was determined in subsequent generations of self-pollination, reciprocal crosses and backcrosses. Thus, the control of the cytoplasmic instability by a single recessive nuclear mutant was also demonstrated [17,18].

The visual spectrum of mutant phenotypes mostly consisted of clonally variegated plants. Chlorophyll deficiencies were sometimes shown together with morphological changes that mainly consisted of shortened and/or narrower leaves. On the other hand, plants carrying aberrant growth patterns or higher levels of sterility were seldom observed, and none of them proved to be inherited. The chlorophyll deficiencies observed in *cpm* plants covered a variable proportion of the leaves, from a very narrow clonal stripe in only one leaf to solid or almost solid chlorophyll-deficient mutant plants. Interestingly, some of the latter were viable until maturity, and thus, they could be genetically stabilized by backcrossing them as mother plants with wild-type pollen. In subsequent generations of auto-pollination, it was possible to select families carrying mutant phenotypes only, without any sign of clonal stripes [18,19]. These families were called cytoplasmic lines (CLs) and carried a genetically stable mutant plastome expressed in the absence of the *cpm* allele. In a few CLs, candidate genes were postulated based on phenotypes and sequenced. Mutations were found in the *infA* [19,20] and the *ycf3* gene [21], as well as in the *psbA* gene in a family isolated after atrazine treatments [22]. In this way, the first data about the occurrence of point mutations in the plastome of *cpm* plants were obtained. They consisted of four substitutions and one insertion.

Using a cpTILLING (chloroplast-targeting induced local lesions in genomes) strategy directed to 33 genes and a few intergenic regions [23], a wide spectrum of molecular changes was detected in the plastome of plants belonging to families that carried the *cpm* genotype through many generations. Numerous polymorphisms, both in genic and intergenic regions, were detected, accounting for at least 61 independent mutational events. Substitutions and small indels (insertions/deletions) in microsatellites constituted the vast majority of the polymorphisms, while increased numbers of recombination events were also observed [23,24,25]. The activity of the *cpm* mutator seems to be specific to the chloroplast genome, although its molecular effects on the mitochondrial genome were, so far, not determined.

The variety of *cpm*-induced polymorphisms was similar to those identified in mutants of the DNA mismatch-repair system (MMR), which is a complex conserved in multiple species, from bacteria to eukaryotes [26]. Most of the MMR proteins are in charge of mismatch recognition [26,27,28,29] and possess anti-recombination activity [28,29,30]. Therefore, mutations in those *Msh* genes were considered prime candidates for causing the plastome instability observed in *cpm* plants. 

Eukaryotic MutS homolog (MSH) proteins bind DNA molecules with mismatches that have arisen during replication errors. There are seven MSH proteins in plants, which can be grouped in the bacterial MutS1-like and the bacterial MutS2-like subfamilies depending on their lineages and functions [30,31]. MSH1, MSH2, MSH3, MSH6 and MSH7 carry mismatch-recognition domains as proteins belonging to the MutS1 subfamily. They also have an anti-recombination activity between divergent sequences [26,30,32,33]. MSH1 forms homodimers, while MSH2 forms heterodimers with MSH3, MSH6 and MSH7. MSH4 and MSH5 proteins belong to the MutS2 subfamily. They form heterodimers that recognize branched DNA structures, such as Holliday junction, are involved in meiotic crossing-over and are lacking mismatch-recognition domains [34,35]. 

In Arabidopsis, MSH1 has been reported as required for maintaining both, mitochondrial [36,37,38,39] and chloroplast DNA stability [2,40], while MSH2, MSH3, MSH6 and MSH7 were found to be required for nuclear genome stability [41,42,43,44]. In bacteria, MutS proteins perform their tasks together with two other MMR proteins, the adaptor protein MutL and the endonuclease MutH [29,45]. The same holds true for plant MSH proteins, with the exception of MSH1. The functions of mismatch recognition and endonuclease activity seem to be included in the peculiar domain architecture of MSH1, which is the only MSH protein containing an endonuclease GIY-YIG domain [31,46]. Therefore, MSH1 is not expected to require proteins homologous to MutL or MutH. This is consistent with the notion that MutL and MutH homologous are not targeted to the organelles [47]. 

In this investigation, seven *Msh* homologous genes were identified, five of which were sequenced in *cpm* and control seedlings. A substitution introducing a premature stop codon in exon 17 of the *cpm Msh*1 gene was found. In addition, the relationship between the *Msh*1 mutant allele and the mutator phenotype was able to be established in progenies from crosses and backcrosses.

Taken together, these results suggest that the *Msh*1 gene mutant is a strong candidate to be responsible for the chloroplast genomic instability induced in *cpm* plants.

## 2. Results

### 2.1. Identification and Amplification of the Coding Sequences of Barley Msh Genes 

Searching for the orthologs of the *Arabidopsis thaliana MSH* genes in barley, six loci with functional annotation were identified: *Msh*1 (HORVU2Hr1G087660), *Msh*2 (HORVU1Hr1G030930), *Msh*3 (HORVU2Hr1G085940), *Msh*4 (HORVU2Hr1G031870), *Msh*5 (HORVU1Hr1G066830) and *Msh*6 (HORVU5HrG1061020). The barley *Msh*7 gene could not be identified; however, the *Msh*7 gene of another cereal (*Triticum turgidum*) was found, and its amino-acidic sequence was used to perform a tblastn against the *Hordeum vulgare* genome. Several hits with different lengths of alignments were obtained for one locus of barley (HORVU3Hr1G021520). In this way, this locus was named as barley *Msh*7 gene because it was understood that it did not have a functional annotation. 

**Figure 1:**
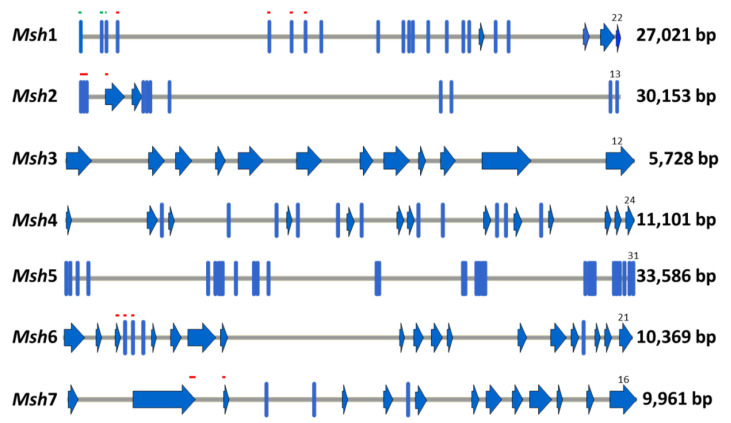
Structural annotation of barley *Msh* genes. The structure was determined by alignment of its encoded proteins with the *A. thaliana* proteins and corroborated by sequencing of cDNAs covering the CDS in *Msh*1, *Msh*2, *Msh*3, *Msh*6 and *Msh*7. Blue lines and arrows represent exons, and grey lines represent introns. The DNA regions encoding the mismatch-recognition domains are indicated by red lines, and the dual-organelle signal peptide by green lines. The region encoding the *Msh*3 mismatch-recognition domain is not shown because it is located in the missing part of this gene in comparison to the *A. thaliana* gene. *Msh*4 and *Msh*5 do not have regions encoding mismatch-recognition domains. The gene sizes and the number of exons are indicated on the right.

The identity of each one of the *Msh* loci in barley was searched for by alignments of the proteins encoded by these genes with the MSH proteins of *A. thaliana*, where the identity positions were higher than 50% (60.5% for MSH1, 64.8% for MSH2, 53% for MSH3 considering only the last part of the *A. thaliana* protein, 73.2% for MSH4, 60.1% for MSH5, 53.6% for MSH6 considering only the last part of the *A. thaliana* protein), with the exception of MSH7, where this value was 42.6%. Moreover, similar structural annotations to *A. thaliana* genes for each of the barley *Msh* genes could be observed. In this way, the most probable structural annotation was determined. The gene structures are shown in Figure 1. Briefly, they corresponded to splicing variants producing some of the isoforms predicted in the database Ensembl Plants for MSH1, MSH2, MSH3 and MSH4, and it was a combination of exons belonging to different predicted isoforms for MSH5, MSH6 and MSH7 (See Table S1). Moreover, in comparison with the *A. thaliana* proteins, barley MSH3 and MSH6 lacked the first part of the protein, and MSH7 had an extra part at the beginning of the protein not present in *A. thaliana* MSH7.

The sizes of the genes ranged from 5.73 Kb for *Msh*3 to 33.59 Kb for *Msh*5 (see Figure 1). Because of these large sizes, it was decided to amplify the coding sequences (CDS) of these genes in barley. The overlapping amplicons designed to cover the complete CDS are listed in Table S1. Amplicons of the estimated sizes were obtained using RT-PCR of all analyzed genes in both *cpm* and control genotypes. This indicates that these genes were expressed in the embryos from which the RNA samples were isolated and also that the structural annotations used for primer design were correct.

Using TargetP software, signal peptides to organelles were identified. With version 1.1, a dual location to mitochondria and chloroplasts was predicted for MSH1. MSH5 and MSH7 had a predicted location in mitochondria only, although with a low confidence value. However, with version 2.0, only the dual organelle localization of the MSH1 protein was confirmed (Table S2A and Table S2B, respectively).

### 2.2. Sequencing and Detection of a Premature Stop-Codon Mutation in the Cpm Msh1 Gene 

**Figure 2:**
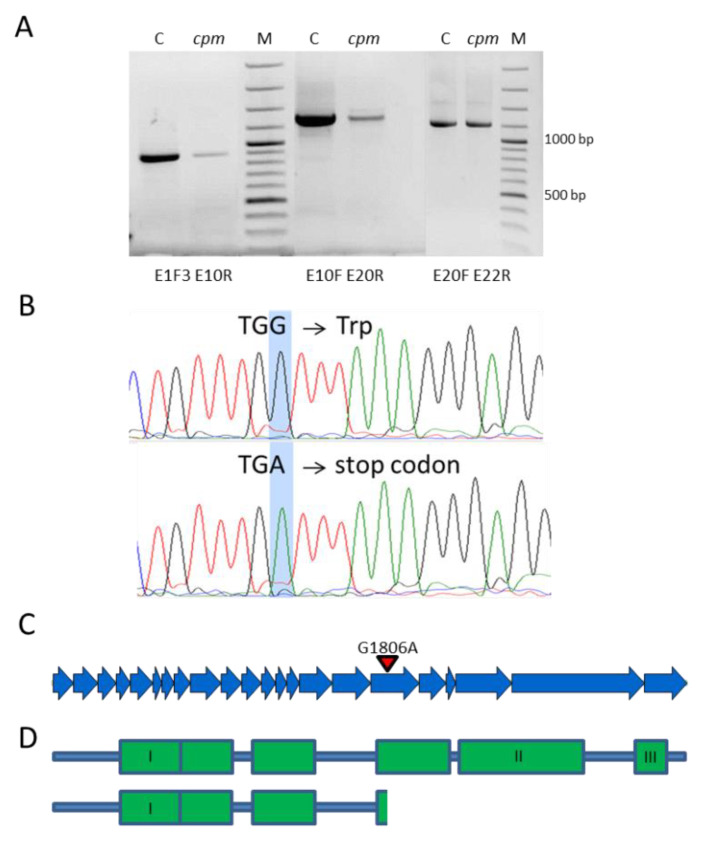
Amplification and sequencing of *Msh*1 gene from *cpm* and control genotypes. (**A**) Amplicons covering the CDS of the *Msh*1 gene obtained by RT-PCR from embryo RNA. C: control, *cpm*: barley chloroplast mutator, M: molecular marker. E1F3E10R, E10FE20R and E20FE22R represent the three CDS amplicons sequenced in the *Msh*1 gene (see Supplementary Materials). (**B**) Electropherograms showing the stop-codon mutation G1806A in *cpm*. (**C**) Location of the substitution G1806A in exon 17 of the *Msh*1 gene in *cpm*. (**D**) Domains of the MSH1 protein in the control (upper) and *cpm* (lower) genotypes. I and II represent the most characteristic domains of MSH proteins. I is the DNA binding domain, II is the ATPase domain and III is the endonuclease GIY-YIG domain (only present in the MSH1 protein).

Comparison of the amplicon sequences covering the CDS of five *Msh* genes of *cpm* and the control genotype with the reference sequence (Ensembl Plants *Hordeum vulgare* IBSC_v2 release-51) confirmed the correct structural annotation used in the primer design for sequencing. *Msh*4 and *Msh*5 were not sequenced because they do not have a mismatch-recognition domain. The *cpm* sequences only differed from that of the control in a single polymorphism, while *cpm* and control presented several polymorphisms with respect to the reference sequence. The sequences of the *Msh*2 and *Msh*3 genes of control and *cpm* were identical to that of the reference. On the other hand, 16 substitutions were identified in the *Msh*1 gene, five in the *Msh*6 and seven in the *Msh*7 genes of *cpm* and control, with respect to the reference. Thirteen of these substitutions would change the amino-acid sequence in the encoded protein, and one of these changes was only found in the *cpm* genotype (see Table 1). As mentioned above, the *cpm* sequence only differed from that of the control in a single polymorphism; it was a G1806A substitution in the *Msh*1 gene that generates a premature stop codon in exon 17. This mutation would produce a truncated protein of half the size of the wild-type version, which would lose the DNA binding and the ATPase domains (Figure 2). Alignment of MSH1 proteins from the reference, *cpm* mutant and the control genotype is shown in Figure S1. The *Msh* CDS sequences of the analyzed genes in *cpm* and the control were deposited in the Genbank database (see Data Availability Statement).

### 2.3. Analysis of the Msh1 DNA Region Carrying the Premature Stop Codon in Cpm and CLs Seedlings

In order to confirm the presence of the premature stop-codon mutation identified in the CDS of the *cpm Msh*1 gene, DNA samples were isolated from five different *cpm* seedlings and five of the control. After sequencing a 655 bp amplicon comprising exon 16, intron 16 and exon 17, the existence of the premature stop-codon mutation was confirmed only in *cpm* seedlings (see electropherograms in Figure 2). 

**Figure 3:**
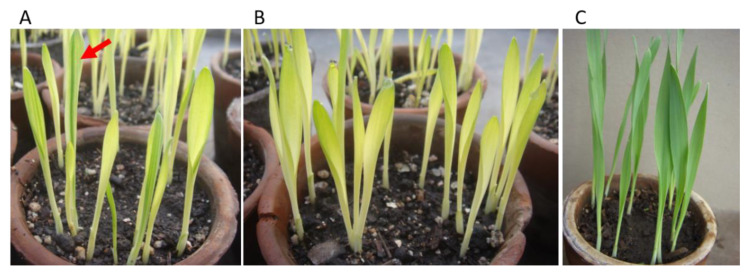
Progenies of CL4 plants carrying *cpm* mutator- or *Cpm* wild-type nuclear genotypes. (**A**) Segregation of solid virescent seedlings or clonally variegated ones (red arrow). Variegated seedlings (*cpm* genotype) show darker green stripes of different sizes on a virescent background, which supposedly originated in the slow and unleashed sorting out of plastome mutants in comparison with the segregation of nuclear ones, which are expected to segregate as solid mutants since the F2 generation. (**B**) Seedlings showing a solid mutant virescent phenotype (*Cpm* genotype). (**C**) Control wild-type seedlings.

With the purpose of confirming the relationship between the premature stop-codon mutation in the *Msh*1 gene and the plastome instability that produces chlorophyll deficiencies in *cpm* seedlings, seven CLs were analyzed. As explained in Materials and Methods, CLs are homoplastomic, each carrying only one particular mutant plastome that gives a determined chlorophyll-deficient type. They did not show any clonal longitudinal stripe with different chlorophyll pigmentation that would indicate they were genetically unstable. In accordance with this, by sequencing the 655 bp amplicon covering exons 16 and 17 of the *Msh*1 gene in seven CLs, it was determined that none of them had the *Msh*1 mutation. In addition, one of those CLs (CL4) was characterized as showing a high rate of phenotypic reversions in segregating progenies from backcrosses of CL4 plants as females with *cpm* pollen [18]. The phenotypic reversions were easily observed as the appearance of darker green stripes on a CL4 virescent background (Figure 3), and this was used to easily identify seedlings that recovered plastome instability as early as in the F_2_ generation. In two families derived from seedlings showing phenotypic reversions, it was observed that they carried the stop-codon mutation in the *Msh*1 gene. A diagram showing the handling of the material to investigate phenotypic reversions in another CL (CL2) can be seen in a previous paper [19]. 

### 2.4. Prediction of Alternative Subcellular Locations of MSH1 Protein in Barley

**Figure 4:**
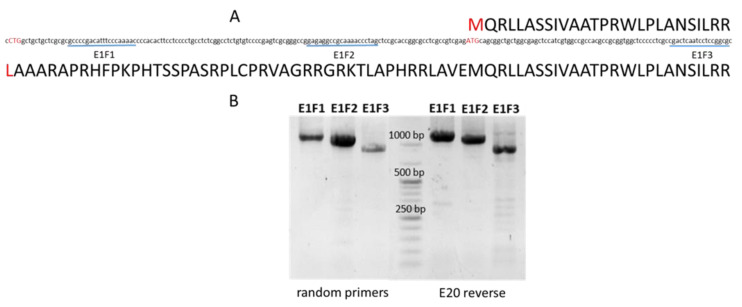
Alternative start codons of the *Msh*1 gene in barley. (**A**) Typical (ATG), alternative and atypical start codon (CTG) on the 5′UTR sequence of the barley *Msh*1 gene are shown in red, along with, the beginning of the MSH1 protein with dual organelle (upper) or chloroplast-only targeting (lower). The positions of three different forward primers (E1F1, E1F2 and E1F3) are indicated by blue lines. (**B**) Amplification of the 5′UTR of barley *Msh*1 transcripts. Three forward primers were used: E1F1 and E1F2, located in the 5′UTR, and E1F3, located downstream of the typical ATG start codon. Control cDNA was obtained using both random primers and E20 reverse primer. Amplicons E1F1-E11R (1196 bp) and E1F2-E11R (1124 bp) were observed, as well as E1F3-E10R (924 bp).

Analysis of the barley *Msh*1 gene 5′UTR sequence showed the presence of the atypical translation initiation codon CTG, apart from the typical ATG codon (Figure 4A). 

To study the existence of mRNA containing the 5′UTR with the atypical start codon, the amplification of this region in the *Msh*1 gene of the control barley was made using forward primers located in three different positions of the gene. E1F1 and E1F2 were located upstream of the typical ATG start codon, and the E1F3 primer was located downstream of the ATG start codon. Amplifications were obtained with all the forward primers, which indicates the presence of the 5′UTR in transcripts of the *Msh*1 gene of barley (Figure 4B).

According to TargetP-1.1, the prediction of the subcellular location of the protein is different, depending on the initiation codon used (Table 2). A dual mitochondrial/chloroplast location was predicted for a protein synthesized from the initiation codon ATG, while only a chloroplast location was predicted if the barley MSH1 protein was translated from the CTG codon. 

## 3. Discussion

### 3.1. A Mutation in the Msh1 Gene as Candidate for the Barley cpm 

The diverse plastome polymorphisms previously observed in barley *cpm* plants exhibited a landscape of molecular changes typical of a missing DNA repair system dedicated to function in the chloroplasts [16,23,24,25]. In this communication, the efforts to identify the repair system, the mutation of which led to the chloroplast mutator *cpm,* are described. Genes coding for mismatch-repair enzymes were identified based in their homology to Arabidopsis genes. Seven genes were identified, and five of them were sequenced; *Msh*3 and *Msh*4 genes were not sequenced since they lack the mismatch-recognition domains. Target-P prediction of subcellular location of barley MSH proteins showed that MSH1 would be the only one with a chloroplast/mitochondrial dual-targeting peptide. Like the Arabidopsis homolog, the *Msh*1 gene of barley consists of 22 exons and is of similar size [36].

The sequence of the *Msh*1 gene of the *cpm* plants exhibited a G1806A mutation in exon 17, leading to a predicted translational stop. This mutation was the only difference found between all sequenced *cpm* and wild-type control *Msh* genes. The premature stop codon would result in a truncated MSH1 protein of half the size of the normal version, which would drastically affect the function of the protein due to the loss of the ATPase and the endonuclease domains. Importantly, in seven CLs carrying a genetically stable plastome (see Materials and Methods), the stop-codon mutation was absent. Thus, the stable mutant chloroplast genotype is independent of the mutated mismatch-repair system. On the other hand, the G1806A mutation was present in plants that recovered the phenotypic instability in the F_2_ and further segregating generations resulting from backcrossing plants of the highly reverting Cytoplasmic Line 4 (CL4) with *cpm* pollen [18]. These results strongly support the hypothesis that the stop-codon mutation in the *Msh*1 gene is responsible for the high frequency and the wide spectrum of chlorophyll-deficient types, as well as for the numerous and varied plastome molecular changes observed in *cpm* plants. 

### 3.2. Barley and Arabidopsis Mutants Exhibit Different Phenotypes

The most commonly reported phenotypes of the Arabidopsis chloroplast mutator (*chm*) mutants were conspicuous white, yellow-green variegation and distorted leaves associated with high levels of sterility [48,49]. These mutant plants exhibited new restriction fragments in the mitochondrial genome due to DNA rearragments [49,50]. The *Chm* gene, renamed *AtMSH*1, was shown to encode a protein related to the *E. coli MutS* gene and the yeast mitochondrial *MSH*1 gene [36]. The *AtMSH1* gene controls substoichiometric shifting, which involves rapid and dramatic changes in relative copy number of portions of the mitochondrial genome over the timespan of one generation. Subgenomic DNA molecules often contain recombination-derived chimeric sequences, and the genomic shifting can alter plant phenotype because the process activates or silences mitochondrial sequences located on the shifted molecule [36]. Mutations in either the ATPase or the endonuclease domains were observed to trigger the mitochondrial substoichiometric shifting, as well as the variegated phenotype [46]. 

While no plastome polymorphisms were found during the first investigations in the *Atmsh*1 mutants [49], a low frequency of plastome recombination events was later observed [51]. Lack of AtMSH1 protein led to a massive increase in the frequency of point mutations and small indels in mitochondrial and the plastid DNA, as was recently shown [2]. Depending on which of the two start codons is used, an AtMSH1 protein translated from a typical ATG codon preferentially targets mitochondria while a protein translated from an atypical start codon exhibits dual targeting [52].

The differential targeting to the organelles, depending on the species, could explain the differences between the *cpm* barley and the Arabidopsis mutants [46,47,52]. In this regard, it is interesting to realize that an alternative start codon could be identified in the barley MSH1 protein. Morever, the presence of transcripts covering the 5′UTR, in which the sequence of the alternative start codon is located, was shown by RT-PCR. TargetP predicts a chloroplast location for the protein translated from the alternative codon. Therefore, the Arabidopsis protein could be mainly involved in mitochondrial DNA stability and the barley homolog in maintaining chloroplast DNA stability. This hypothesis requires further investigation to assess the possible influence of the barley MSH1 protein on the mitochondrial genome. 

In the *cpm* mutant, variegation was not associated with high levels of sterility shown for Arabidopsis *msh*1 mutants [49]. Additionally, effects of developmental reprogramming due to depletion of the Arabidopsis MSH1 protein [53] were not observed in *cpm* plants [17,18,23]. This was observed in several plant species and was related to epigenetic modifications in nuclear genes [54,55,56,57,58,59].

Another reason for the differences observed between the mutants in Arabidopsis and barley could be based on the different kinds of mutations these plants carry. Arabidopsis knockout mutants [2,36,51] or the RNAi mutants obtained in multiple species [51] may have more drastic consequences than those of the premature stop-codon mutation identified in *cpm* plants. In this context, it should be considered that the barley mutant is expected to retain the mismatch-recognition function of the MSH1 protein. Similarly, an Arabidopsis mutant carrying a T-DNA insertion in the eighth intron of the MSH1 gene could also reduce but not eliminate its function, resulting in weaker phenotypic effects and lower mutation rates than those induced by the knockouts [2]. 

## 4. Materials and Methods

### 4.1. Plant Material

Plant material consisted of embryos and seedlings of a two-row cultivated barley (*H. vulgare*) genotype, homozygous for the barley chloroplast mutator (*cpm*) gene, using as control its parental line from which it was isolated after mutagenic treatments [17]. Besides, plant material included seedlings belonging to seven cytoplasmic lines (CLs): CL1, CL2, CL3, CL4, CL5, CL7c and CL13. Each CL has one particular chlorophyll mutant phenotype that was induced by the *cpm*. The CLs were obtained after crossing *cpm* chlorophyll-deficient plants as females with control plants and later selecting among the segregating families those not showing new stripes of different colors than that of the mutant background. For this reason, they are considered to be carrying a genetically stable cytoplasm. Obtainment of the CLs and description of the phenotypes of some of the CLs were already reported [16,18,19,20,21,60]. One of them, CL4, is characterized by a virescent phenotype (yellowish young leaves that gradually turn to normal or almost normal green at maturity), which shows a high rate of phenotypic reversions in segregating progenies coming from backcrosses of CL4 as female with *cpm* pollen [18]. This CL4 peculiarity was used to identify seedlings that recovered the plastome instability as early as in the F_2_ generation. 

Embryos of *cpm* and the control genotype were used for RNA isolation after 48 h of germination in Petri dishes. DNA samples were extracted from the second leaf of *cpm*, control and CLs seedlings grown in pots under glasshouse conditions. 

### 4.2. Identification of Msh Genes of Barley

The reference sequences of *Msh* genes were obtained from the *H. vulgare* database of Ensembl Plants (https://plants.ensembl.org/index.html (accessed on 19 June 2021)) as orthologous of *MSH* genes of *A. thaliana*. The most probable structural annotation (exons, introns and CDS sequences) of barley *Msh* genes was determined by alignment of the protein sequences between *A. thaliana* and all the possible isoforms of barley MSH proteins. Besides, predictions for their subcellular location for each of the MSH barley proteins were obtained by identification of N-terminal signal peptides using TargetP-1.1 and -2.0 versions (Technical University of Denmark, Lyngby, Denmark; http://www.cbs.dtu.dk/services/TargetP/ (accessed on 09 July 2021)).

### 4.3. Amplification and Sequencing of the Coding Sequences of Msh Barley Genes 

The exon sequences of five *Msh* genes in *cpm* and the control were obtained by Sanger sequencing the copy DNA from embryo RNA. Primers (see Table S1) were designed with Primer3 software (ELIXIR—European research infrastructure for biological information, University of Tartu, Tartu, Estonia; https://primer3.ut.ee/ (accessed on 21 August 2021)) using the most probable structural annotation of barley *Msh* genes. Moreover, the 5′UTR region of the *Msh*1 gene was amplified using three different forward primers: E1F1 and EIF2 (5′-GCCCCGACATTTCCCAAAAC-3′ and 5′-GAGAGGCCGCAAAACCCTAG-3′) in combination with E11R (5′-GCCTCCCTTCCAACGAGATAG-3′) and E1F3 (5′-CGACTCAATCCTCCGGCG-3′) in combination with E10R (5′-GAACCATCAAACCAGTCAAAGGA-3′). The amplification of a single band each of the sizes expected for these amplicons was determined by RT-PCR before sequencing. For the RT-PCR, approximately 100–200 mg of embryos was collected from seeds grown for 48 h with an 8 h photoperiod, flash frozen with liquid nitrogen and processed using Trizol (Invitrogen, Waltham, MA, USA) for total RNA isolation without DNase digestion. RNA concentrations were measured using a spectrophotometer (Nanodrop, Thermo Scientific, Wilmington, DE, USA). cDNA was synthesized with Superscript III reverse transcriptase using random primers and 1 μg of RNA, according to the manufacturer’s instructions (Invitrogen, Waltham, MA, USA). PCRs for amplifying the cDNA were performed using 2 μL of cDNA, 1× Taq buffer, 1.25 mM MgCl_2_, 0.6 µM of each primer, 0.6 mM of deoxynucleotide triphosphate (dNTP) mix and 1.25 units kit T-plus 5 U/µL Taq DNA polymerase (Inbio Highway, Tandil, Argentina). After denaturation at 94 °C for 3 min, the reaction mixtures were heated to 94 °C for 30 s, 60 °C for 1 min, and 72 °C for 2 min in 35 cycles and 10 min at 72 °C.

### 4.4. Analyses of DNA Sequences of cpm, Control and CLs Seedlings

A genomic DNA region corresponding to the *Msh*1 gene was amplified and sequenced in *cpm*, control and several CLs. Genomic DNA was isolated from 1 or 2 leaves of individual seedlings using the micro method described in [61] with modifications. The tissue was ground with Dellaporta isolation buffer in the The Fast Prep^®^-24 Instrument (MP Biomedicals, Santa Ana, CA, USA) and extracted with chloroform before DNA precipitation. DNA concentrations were measured using a spectrophotometer (NanoDrop, Thermo Fisher Scientific, Wilmington, DE, USA) and standardized to a concentration of 80 ng/µL. A PCR reaction was performed in a final volume of 12.5 µL using 40 ng of genomic DNA, 1× Taq buffer (50 mM KCl, 10 mM Tris–HCl, pH 9.0, 0.1% Triton X-100), 1.25 mM MgCl_2_, 0.6 µM of each primer, 0.6 mM dNTP mix and 1.25 units kit T-plus 5 U/µL Taq DNA polymerase (Inbio Highway, Tandil, Argentina). After denaturation at 94 °C for 3 min, the reaction mixtures were heated to 94 °C for 30 s, 60 °C for 1 min and 72 °C for 1 min in 35 cycles and 10 min at 72 °C. A 655 bp fragment was amplified using primers Hvmsh1_914F 5′-ACCAGGCAATATCTTCATCGGA-3′ and Hvmsh1_914R 5′-ACCATTCTTCCCCAACCCTTC-3′.

### 4.5. Alignments and Polymorphism Identification

cDNA, DNA and protein sequences of *cpm*, control and CLs were aligned with Clustal O 1.2.4 Software (EMBL-EBI, Cambridge, United Kingdom) and Vector NTI 10.0. Software (Life Technologies, Carlsbad, CA, USA) for polymorphisms identification. Assembly and graphics were done with Vector NTI 10.0. software. 

## Figures and Tables

**Table 1:** Polymorphisms identified in *Msh* genes of *cpm* and control genotypes in comparison with the reference sequence (Ensembl Plants *Hordeum vulgare* IBSC_v2 release-51). In *Msh*2 and *Msh*3, no changes were found. *Msh*4 y *Msh*5 were not sequenced because they do not have a mismatch-recognition domain.

Gene	Nucleotide Change ^a^	Exon Location	Amino-Acid Change	Genotype
*Msh*1	A216G	2	-	*cpm* and control
	T225C	2	-	*cpm* and control
	A263C	3	Q88P	*cpm* and control
	A300G	3	-	*cpm* and control
	T696C	8	-	*cpm* and control
	T831C	9	-	*cpm* and control
	G1806A	17	W602 *	*cpm*
	T2060C	18	M687T	*cpm* and control
	G2799A	21	-	*cpm* and control
	G2861A	21	R954Q	*cpm* and control
	C2988T	21	-	*cpm* and control
	G3000A	21	-	*cpm* and control
	G3046A	21	V1016I	*cpm* and control
	G3135T	21	-	*cpm* and control
	C3226T	22	-	*cpm* and control
	A3235G	22	M1079V	*cpm* and control
*Msh*6	G498A	3	-	*cpm* and control
	T1144C	9	-	*cpm* and control
	T1577C	10	L526S	*cpm* and control
	A2340G	15	-	*cpm* and control
	G2464A	16	V822I	*cpm* and control
*Msh*7	C337T	2	P113S	*cpm* and control
	T440C	2	V147A	*cpm* and control
	T1739C	7	F580S	*cpm* and control
	G1860T	9	M620I	*cpm* and control
	A3030G	14	-	*cpm* and control
	C3171T	15	-	*cpm* and control
	G3317A	16	R1106K	*cpm* and control

^a^ coordinate refers to the coding sequence (CDS). * indicates stop codon.

**Table 2:** Prediction of the subcellular locations of barley MSH1 protein versions according to TargetP-1.1 software, Technical University of Denmark, Lyngby, Denmark. The location with the highest score is the most likely one according to TargetP.

Protein	Mitochondrial Transfer Peptide	Chloroplast Transfer Peptide	Signal Peptide to Secretory Pathway	Any Other Location	Location
MSH1 typical start codon	0.791	0.620	0.004	0.006	Mitochondria Chloroplast
MSH1 alternative start codon	0.279	0.754	0	0.098	Chloroplast

## Data Availability

The *Msh* CDS sequences of the analyzed genes in *cpm* and the control were deposited in the Genbank database: *cpm Msh*1 (Genbank accession no. MZ747162), control *Msh*1 (Genbank accession no. MZ747163), *cpm Msh*2 (Genbank accession no. MZ747164), control *Msh*2 (Genbank accession no. MZ747165), *cpm Msh*3 (Genbank accession no. MZ747166), control *Msh*3 (Genbank accession no. MZ747167), *cpm Msh*6 (Genbank accession no. MZ747168), control *Msh*6 (Genbank accession no. MZ747169), *cpm Msh*7 (Genbank accession no. MZ747170) and control *Msh*7 (Genbank accession no. MZ747171).

## Supplementary Materials

The following are available online at https://www.mdpi.com/article/10.3390/ijms23031814/s1.
