# Supplementary material for: The Barley Chloroplast Mutator (cpm) Mutant: All Roads Lead to the Msh1 Gene
Source: Int J Mol Sci. 2022 Feb 5;23(3):1814. doi: 10.3390/ijms23031814 (PMC8836938; doi:10.3390/ijms23031814)
Supplement: Supplementary file 1 [file ijms-23-01814-s001.zip › ijms-1369283-supplementary.pdf]

Supplementary Materials

Table S1. List of primers and amplicons for sequencing the coding regions of the barley *Msh* genes.

Gene/<br>Locus-Amplicon	Forward Primer	Reverse Primer	Amplicon Size (bp)
Msh1/<br>HORVU2Hr1G087660.5 [a]	E1F3<br>CGACTCAATCCTCCGGCG	E10R<br>GAACCATCAAACCAGTCAAAGGA	924
	E10F<br>TCTTGTGGGGAGAGTGCAAC	E20R<br>AGCGCATACTGACATCGTTCA	1326
	E20F<br>TGCAGGATTATAGTACTGAAGAAAGC	E22R<br>TGGCTTCTCCAGAGGTTACAG	1222
Msh2/<br>HORVU1Hr1G030930.2 [a]	E1F<br>TTTGCACACATTTCGCCGAG	E6R<br>AGCACAGCCAAATCAGGAGA	1496
	E6F<br>ACAGCCATTGACCTCGATCA	E13R<br>GGGATTACTGGCTGCGTCTT	1452
Msh3/<br>HORVU2Hr1G085940.3 [a,e]	E1F<br>CCAGAAGAGGGGAGCAGGTA	E7R<br>TGGTAGTTCGATCAGGTAAGCTG	1302
	E6F<br>GCTGACCAGTTCCCCGAG	E12R<br>AGCTTTCCCGTTGTCCATCC	1249
Msh4/<br>HORVU2Hr1G031870.3 [a]	It was not amplified nor sequenced		
Msh5/<br>HORVU1Hr1G066830 [b]	It was not amplified nor sequenced		
Msh6/<br>HORVU5HrG1061020 [c,e]	E1F1<br>CTGTGCAGGAGGATGAGGAT	E8R<br>TTGAAAATAGCAATGGCACCT	1669
	E8F1<br>TGAATCATTGTGTTACTGGATTTG	E13R1<br>GACACCAACCGTCTCCACTT	838
	E13F1<br>CTGCCGGTACTGGACACC	E17R1<br>CCTGGCACGCATCTGTACT	1114
Msh7/<br>HORVU3Hr1G021520 [d]	E1F1<br>CAGCAGCAGTCCATCCTCTC	E5R<br>CCTCTAACTTTCGCCTGTGC	1460
	E5F<br>ATGGAATCTGCAGCACAGG	E12R<br>TCATCAAGAGAGCTCAGGGTTG	1085
	E11F<br>CTTGGACGAGTCAGATCCAC	E16R1<br>TGGGCGACACAGAACATC	1313

a. The numbers after the dots correspond to the splice variant producing the most probable protein isoform, which was used to design the primers.
b. In this locus, the splice variant producing the most probable protein isoform does not correspond to any of the variants shown in Ensembl Plants database. Actually, it is a mixture of those producing isoforms 28 and 9.
c. In this locus, the splice variant producing the protein isoform that was used to design the primers does not correspond to any of the variants shown in Ensembl Plants database. Actually, it is a mixture of those producing isoforms 32 and 11.
d. In this locus, the splice variant producing the protein isoform that was used to design the primers does not correspond to any of the variants shown in Ensembl Plants database. Actually, it is a mixture of those producing isoforms 35 and 20.
e. The proteins encoded in these loci lack the first part in comparison to the *A. thaliana* protein.

Table S2A. Prediction of the subcellular location of barley MSH proteins with TargetP-1.1.

Protein	Mitochondrial transfer peptide	Chloroplast transfer peptide	Signal peptide to secretory pathway	Any other location	Location
MSH1	0.791	0.620	0.004	0.006	Mitochondria Chloroplast
MSH2	0.122	0.068	0.044	0.952	-
MSH3*	0.122	0.041	0.030	0.968	-
MSH4	0.175	0.103	0.051	0.865	-
MSH5	0.503	0.020	0.232	0.499	Mitochondria
MSH6*	0.045	0.075	0.025	0.939	-
MSH7	0.571	0.299	0.008	0.076	Mitochondria

*These proteins lack the first part in comparison to the *A. thaliana* protein.

Table S2B. Prediction of the subcellular location of barley MSH proteins with TargetP-2.0.

Protein	Mitochondrial transfer peptide	Chloroplast transfer peptide	Thylakoid luminal transfer peptide	Signal peptide to secretory pathway	Any other location
MSH1	0.4146	0.3796	0.0023	0.0240	0.2010
MSH2	0	0	0	0	1
MSH3*	0	0	0	0.0001	0.9999
MSH4	0	0	0	0.0002	0.9998
MSH5	0.0001	0	0	0.0005	0.9994
MSH6*	0	0	0	0	1
MSH7	0.0001	0	0	0.0005	0.9985

*These proteins lack the first part in comparison to the *A. thaliana* protein.

Figure S1. Alignment of *H. vulgare*, *cpm* and control MSH1 proteins. Mismatch-recognition, ATPase and endonuclease domains are indicated by red, blue and green boxes, respectively.

```
H. vulgare    MQRLLASSIVAATPRWLPLADSILRRRRPRRSPLPILLFNRSWSKPTKVSRSISMVSSKA  60
control       MQRLLASSIVAATPRWLPLADSILRRRRPRRSPLPILLFNRSWSKPTKVSRSISMVSSKA  60
cpm           MQRLLASSIVAATPRWLPLADSILRRRRPRRSPLPILLFNRSWSKPTKVSRSISMVSSKA  60
              ************************************************************

H. vulgare    NKQGDLCNEGMLSHIMWWKERMESCRKQSSVQLTQRLVYSNILGLDSTLRNGSLKDGTLN  120
control       NKQGDLCNEGMLSHIMWWKERMESCRKPSSVQLTQRLVYSNILGLDSTLRNGSLKDGTLN  120
cpm           NKQGDLCNEGMLSHIMWWKERMESCRKPSSVQLTQRLVYSNILGLDSTLRNGSLKDGTLN  120
              *************************** ********************************

H. vulgare    MEMLQFKSKFPREILLCRVGDFYEAIGFDACILVEHAGLNPFGGLRSDSIPKAGCPIMNL  180
control       MEMLQFKSKFPREILLCRVGDFYEAIGFDACILVEHAGLNPFGGLRSDSIPKAGCPIMNL  180
cpm           MEMLQFKSKFPREILLCRVGDFYEAIGFDACILVEHAGLNPFGGLRSDSIPKAGCPIMNL  180
              ************************************************************

H. vulgare    RQTLDDLTRCGYSVCIVEEIQGPTQARARKGRFISGHAHPGSPYVFGLAEVDHDLEFPDP  240
control       RQTLDDLTRCGYSVCIVEEIQGPTQARARKGRFISGHAHPGSPYVFGLAEVDHDLEFPDP  240
cpm           RQTLDDLTRCGYSVCIVEEIQGPTQARARKGRFISGHAHPGSPYVFGLAEVDHDLEFPDP  240
              ************************************************************

H. vulgare    MPVVGISRSAKGYCLISVLETMKTYSAEEGLTEEAVVTKLRICRYHHLYLHSSLRNNSSG  300
control       MPVVGISRSAKGYCLISVLETMKTYSAEEGLTEEAVVTKLRICRYHHLYLHSSLRNNSSG  300
cpm           MPVVGISRSAKGYCLISVLETMKTYSAEEGLTEEAVVTKLRICRYHHLYLHSSLRNNSSG  300
              ************************************************************

H. vulgare    TSRWGEFGEGGLLWGECNGKSFDWFDGSPIDELLCKVREIYGLDEKTSFRNVTISLEGRP  360
control       TSRWGEFGEGGLLWGECNGKSFDWFDGSPIDELLCKVREIYGLDEKTSFRNVTISLEGRP  360
cpm           TSRWGEFGEGGLLWGECNGKSFDWFDGSPIDELLCKVREIYGLDEKTSFRNVTISLEGRP  360
              ************************************************************

H. vulgare    QPLYLGTATQIGVIPTEGIPSLPKMLLPPNCAGLPSMYIRDLLLNPPSFDVASAIQEACR  420
control       QPLYLGTATQIGVIPTEGIPSLPKMLLPPNCAGLPSMYIRDLLLNPPSFDVASAIQEACR  420
cpm           QPLYLGTATQIGVIPTEGIPSLPKMLLPPNCAGLPSMYIRDLLLNPPSFDVASAIQEACR  420
              ************************************************************

H. vulgare    LMCSITCSIPEFTCIPSAKLVKLLESKEVNHIEFCRIKNVLDEIMLMNGNTELSAIQNKL  480
control       LMCSITCSIPEFTCIPSAKLVKLLESKEVNHIEFCRIKNVLDEIMLMNGNTELSAIQNKL  480
cpm           LMCSITCSIPEFTCIPSAKLVKLLESKEVNHIEFCRIKNVLDEIMLMNGNTELSAIQNKL  480
              ************************************************************

H. vulgare    LEPASVVTGLKVDADILIKECRFISKRIGEVISLAGESDQAISSSEYIPKEFFNDMESSW  540
control       LEPASVVTGLKVDADILIKECRFISKRIGEVISLAGESDQAISSSEYIPKEFFNDMESSW  540
cpm           LEPASVVTGLKVDADILIKECRFISKRIGEVISLAGESDQAISSSEYIPKEFFNDMESSW  540
              ************************************************************

H. vulgare    KGRVKRVHAEEEFSNVDVAAQALSTAVTEDFLPIIVRVKSVMSSHGSSKGEISYAKEHGA  600
control       KGRVKRVHAEEEFSNVDVAAQALSTAVTEDFLPIIVRVKSVMSSHGSSKGEISYAKEHGA  600
cpm           KGRVKRVHAEEEFSNVDVAAQALSTAVTEDFLPIIVRVKSVMSSHGSSKGEISYAKEHGA  600
              ************************************************************

H. vulgare    VWFKGRRFTPNVWANTPGEEQIKQLKPAIDSKGRRVGEEWFTTTKVENALARYHEACDNA  660
control       VWFKGRRFTPNVWANTPGEEQIKQLKPAIDSKGRRVGEEWFTTTKVENALARYHEACDNA  660
cpm           V-----------------------------------------------------------  601
              *

H. vulgare    KGKVLELLRGLSSELQDKINILVFCSMLLIITKALFGHVSEGLRRGWVLPAIYPLSKDYS  720
control       KGKVLELLRGLSSELQDKINILVFCSTLLIITKALFGHVSEGLRRGWVLPAIYPLSKDYS  720
cpm           ------------------------------------------------------------  601

H. vulgare    TEESSSEMDLLGLFPYWLNTNQGNAILNDVSMRSLFILTGPNGGGKSSMLRSVCAAALLG  780
control       TEESSSEMDLLGLFPYWLNTNQGNAILNDVSMRSLFILTGPNGGGKSSMLRSVCAAALLG  780
cpm           ------------------------------------------------------------  601

H. vulgare    VCGLMVPAASAVIPHFDSIMLHMKAYDSPADGKSSFQIEMSEIRSLVSRATGRSLVLIDE  840
control       VCGLMVPAASAVIPHFDSIMLHMKAYDSPADGKSSFQIEMSEIRSLVSRATGRSLVLIDE  840
cpm           ------------------------------------------------------------  601

H. vulgare    ICRGTETAKGTCIAGSIIERLDDAGCLGIVSTHLHGIFDLPLSLNNTDFKAMGTEVVNGY  900
control       ICRGTETAKGTCIAGSIIERLDDAGCLGIVSTHLHGIFDLPLSLNNTDFKAMGTEVVNGY  900
cpm           ------------------------------------------------------------  601

H. vulgare    IQPTWRLMDGICRESLAFQTARKEGMPDLIIKRAEELYLNMSRNNKHTSLTLHRPLVANS  960
control       IQPTWRLMDGICRESLAFQTARKEGMPDLIIKRAEELYLNMSRNNKHTSLTLHQPLVANS  960
cpm           ------------------------------------------------------------  601

H. vulgare    SVNGGLVDRPDGLGNGLEPPTGSFGLLRKDVESIVTAICEDKLLDLYNKRSISEQVEVVC  1020
control       SVNGGLVDRPDGLGNGLEPPTGSFGLLRKDVESIVTAICEDKLLDLYNKRSISEQIEVVC  1020
cpm           ------------------------------------------------------------  601

H. vulgare    VTVGAREQPPPSTVGRSSIYIIIRRDNKLYVGQTDDLVGRLGAHRSKEGMQDATILYIMV  1080
control       VTVGAREQPPPSTVGRSSIYIIIRRDNKLYVGQTDDLVGRLGAHRSKEGMQDATILYIVV  1080
cpm           ------------------------------------------------------------  601

H. vulgare    PGKSVACQLETLLINQLPSKGFKLTNKADGKHRNFGMSVTSGEAMAAH      1128
control       PGKSVACQLETLLINQLPSKGFKLTNKADGKHRNFGMSVTSGEAMAAH      1128
cpm           ------------------------------------------------      601
```
